# Total Triterpenoid Extraction from *Inonotus Obliquus* Using Ionic Liquids and Separation of Potential Lactate Dehydrogenase Inhibitors via Ultrafiltration High-Speed Countercurrent Chromatography

**DOI:** 10.3390/molecules26092467

**Published:** 2021-04-23

**Authors:** Yueqi Wang, Liping Guo, Chunming Liu, Yuchi Zhang, Sainan Li

**Affiliations:** 1 Faculty of Chemistry, Northeast Normal University, No. 5268 Renmin Street, Nanguan District, Changchun 130024, China; wanglq022@nenu.edu.cn; 2 China Central Laboratory, Changchun Normal University, No. 677 North Changji Road, Erdao District, Changchun 130032, China; ccsf777@163.com (C.L.); zhangyuchi2001@163.com (Y.Z.); sainan_85@163.com (S.L.)

**Keywords:** *Inonotus obliquus*, ultrafiltration, liquid chromatography, ultrasonic-assisted extraction, lactate dehydrogenase inhibitor, myocardial infarction

## Abstract

Extracts of the fungus *Inonotus obliquus* exhibit cytotoxic properties against different cancers; hence, this fungal species has been extensively studied. This study aimed to extract total triterpenoids from *Inonotus obliquus* using ionic liquids (ILs) and separate potential lactate dehydrogenase (LDH) inhibitors via ultrafiltration (UF)-high-speed countercurrent chromatography (HSCCC). Total triterpenoids from *Inonotus obliquus* were extracted by performing a single-factor experiment and employing a central composite design via ultrasonic-assisted extraction (UAE) and heat-assisted extraction (HAE). The extract was composed of 1-butyl-3-methylimidazolium bromide as the IL and methanol as the dispersant. Ultrafiltration-liquid chromatography (UF-LC) was used to rapidly scan the LDH inhibitors and betulin and lanosterol were identified as potential inhibitors. To obtain these target compounds, betulin and lanosterol with the purities of 95.9% and 97.8% were isolated from HSCCC within 120 min. Their structures were identified using several techniques, among which IL-HAE was fast and effective. This study reports the extraction of triterpenoids from *Inonotus obliquus* by IL for the first time. Collectively, the findings demonstrate that UF-LC is an effective tool for screening potential LDH inhibitors from crude extracts of *I. obliquus* and may help to identify bioactive substances against myocardial infarction, whereas high-purity compounds can be separated via UF-HSCCC.

## 1. Introduction

The fungus *Inonotus obliquus* is mainly distributed in northeastern China, northern Russia, and other cold regions [1,2]. The sclerotium of its fruiting body is nodular (sterile block), sessile, 25–40 cm in diameter, dark gray in appearance, irregularly furrow-marked with a deep crack, and hard and brittle when dry [3]. This fungus can be used as food and medicine for preventing and treating malignant tumors, diabetes, liver diseases, vascular diseases, and acquired immune deficiency syndrome [4,5]. Therefore, *I. obliquus* has attracted considerable attention from researchers owing to its potential anti-cancer properties. For instance, Baek et al. evaluated its cytotoxic activity against four human lung adenocarcinoma cell lines, each with different p53 statuses. They found that the triterpenoids from *I. obliquus* have potential therapeutic effects against lung cancer and revealed the molecular basis of their cytotoxic activity against human lung cancer [6]. Furthermore, Kou et al. studied the anti-inflammatory effect of triterpenoids from *I. obliquus* and reported that all lanostanoids remarkably inhibited nitric oxide (NO) production in lipopolysaccharide-stimulated BV-2 microglial cells [7]. In another study, it has been shown that triterpenoids from *I. obliquus* can confer protection to SH-SY5Y cells against oxidative damage by inhibiting cell apoptosis and by exerting neuroprotective activity against H_2_O_2_ induced cell damage [8].

Triterpenoids are substances with several (generally six) condensed isoprene formed by hydroxyl removal and are essential products of isoprene metabolism [9]. Although most of these are composed of 30 carbon atoms, some have only 27 carbon atoms. These substances are abundant in nature. Triterpenoids, mostly lanoline-type triterpenoids that are synthesized from cyclosqualene with a chair boat chair conformation and often containing the same lanosterol mother nucleus structure, are among the main active components in *I. obliquus* [6,10,11]. Traditional extraction methods such as the organic reagent extraction method are costly and time-consuming [12]. To overcome the limitations of traditional methods, the required amount of organic solvents and extraction time should be reduced, and extraction efficiency should be improved.

Ionic liquids (ILs), also known as “green solvents,” have broad-spectrum solubilities and are not volatile, making them promising alternatives to organic solvents [13]. ILs also have good thermal stability and are tasteless, and can be easily recycled [14]. Furthermore, the physical and chemical properties of ILs vary widely depending on their chemical structure [15], and because of numerous possibilities of ILs structures by cation and anion selection, they are widely used in organic biocatalytic syntheses, clean fuel production, and in other applications [16]. However, several studies have shown that ILs can threaten human health and exert adverse environmental effects [17], and their toxicity varies with their structure and physical and chemical properties [18]. Therefore, ILs should be designed with caution considering their structure–ecotoxicity relationships [15]. In the present study, different ILs, and dispersants at varying solid: liquid ratios, concentration of the extraction solution, and extraction times were assessed for triterpenoid extraction from *I. obliquus*.

Ultrafiltration-liquid chromatography (UF-LC) is particularly advantageous for screening and extracting bioactive compounds because of its small sample size requirement and good reproducibility [19,20]. After separation of ligand-receptor complexes from unbound compounds via UF, ligands can be identified by liquid chromatography-mass spectrometry (LC-MS). High-speed countercurrent chromatography (HSCCC) is a liquid-liquid partition chromatography technology widely used to separate and purify natural products [21]. Owing to its stability, good reproducibility, and non-requirement of a solid carrier, the adsorption phenomenon because of a carrier is eliminated. In the present study, HSCCC was used to separate potential lactate dehydrogenase (LDH) inhibitors from *I. obliquus* [12,21].

## 2. Results and Discussion

### 2.1. Single-Factor Experiments

#### 2.1.1. Differences in Total Triterpenoid Content Extracted Using Seven IL Types

**Figure 1:**
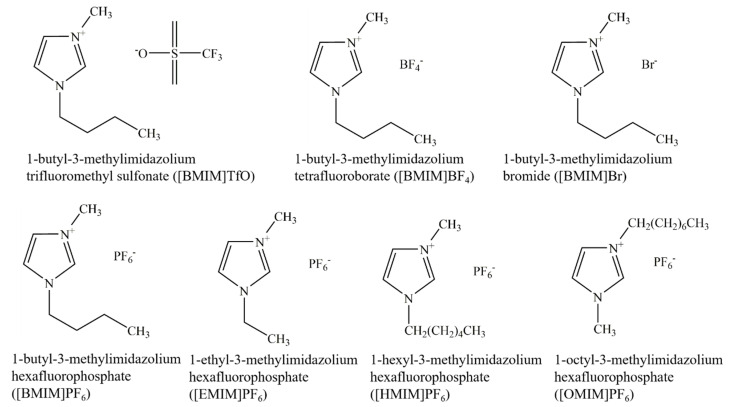
Chemical structures of the seven types of ionic liquids.

Because ILs are composed of cations and anions, the length of the alkyl chain and the composition of anions influence their physical and chemical properties and the extraction efficiency of analytes. Methanol (MeOH) was selected as the dispersant to study the effect of seven ILs (Figure 1) on the total triterpenoids of *I. obliquus*. Four types of imidazole ILs with different carbon-chain lengths (1-ethyl-3-methylimidazolium hexafluorophosphate ([EMIM]PF_6_), 1-butyl-3-methylimidazolium hexafluorophosphate ([BMIM]PF_6_), 1-hexyl-3-methylimidazolium hexafluorophosphate ([HMIM]PF_6_), and 1-octyl-3-methylimidazolium hexafluorophosphate ([OMIM]PF_6_)) and ILs with similar cation and anion compositions (1-butyl-3-methylimidazolium trifluoromethyl sulfonate ([BMIM]TfO, 1-butyl-3-methylimidazolium tetrafluoroborate ([BMIM]BF_4_), 1-butyl-3-methylimidazolium bromide ([BMIM]Br), and [BMIM]PF_6_) were investigated.

**Figure 2:**
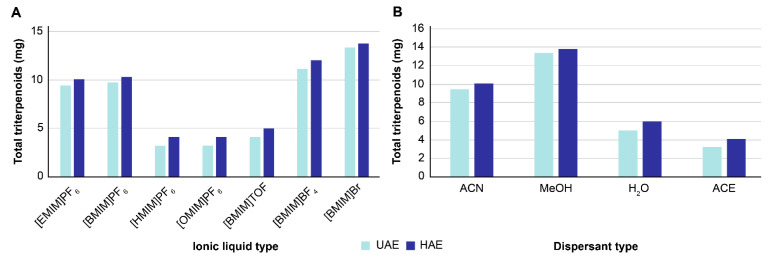
(**A**) Effect of different ionic liquid (IL)-methanol extracts on total triterpenoids. (**B**) Effect of different dispersants on total triterpenoids, with IL = [BMIM] Br.

Under the same extraction conditions, the amounts of total triterpenoids obtained after performing ultrasonic-assisted extraction (UAE) were as follows: 9.45 mg [EMIM]PF_6_, 9.73 mg [BMIM]PF_6_, 3.21 mg [HMIM]PF_6_, and 3.18 mg [OMIM]PF_6_. The amounts of total triterpenoids obtained after performing heat-assisted extraction (HAE) were as follows: 10.12 mg [EMIM]PF_6_, 10.31 mg [BMIM] PF_6_, 4.10 mg [HMIM] PF_6_, and 4.05 mg [OMIM] PF_6_ (Figure 2A). These data show that when anions are the same, the total triterpenoid content first increases and then decreases with increasing carbon chain length regardless of using UAE or HAE. This may be because with increasing carbon chain length, the viscosity of ILs gradually increases, and the effective components cannot be extracted by using the overly viscous ILs. The extraction effect of [BMIM] PF_6_ was the strongest; hence, by using the same cation ([BMIM]^+^), the extraction effect of different anions (TfO^−^, BF_4_^−^, Br^−^, PF_6_^−^) was studied. The results showed that [BMIM] Br exerted the best extraction effect (Figure 2A).

#### 2.1.2. Effect of Dispersant Type on Total Triterpenoid Content

[BMIM] Br was selected to investigate the effect of different dispersants, namely, ACN, MeOH, H_2_O, and ACE, on the extraction capacity. The total triterpenoid content using these dispersants and UAE was 9.45, 13.40, 5.01, and 3.25 mg, respectively. Using HAE, the total triterpenoid content was 10.11, 13.80, 6.02, and 4.11 mg, respectively. The extraction capacity was the highest with [BMIM] Br as IL and MeOH as a dispersant (Figure 2B). Therefore, this IL/dispersant combination was used for follow-up experiments.

### 2.2. Effects of the Central Composite Design

According to the principles of the “central composite test reagent, the experiments were arranged in the form of codes; the codes were obtained based on the actual operation value, and the difference between any two physical quantities was equal to the difference between the corresponding codes. By establishing relevant model fitting effects and factors, the three-dimensional effect surface could be visualized, and the best extraction process was quickly obtained based on the surface area with the best effect.

**Figure 3:**
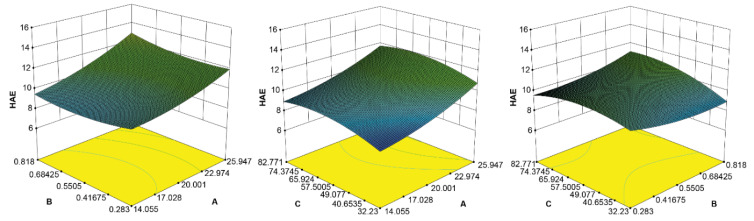
Response surface of the effect of solid: liquid ratio, concentration of the extraction solution (mol/L), and extraction time (min) on total triterpenoids. HAE: heat-assisted extraction; A: solid: liquid ratio; B: concentration of the extraction solution; C: extraction time.

Experimental results of the central composite design using two extraction methods are shown in Table 1. The three factors (A, solid-liquid ratio; B, concentration of the extraction solution; and C, extraction time) were fitted using the total triterpenoid content as the evaluation index. The fitting equations of UAE and HAE are shown in Table 2. The fitting degree of each equation was good (high R^2^ values), and thus, they were used to analyze and predict the extraction process. The results of the binomial fitting analysis of variance (Table 2) showed that the total model equation was significant (*p* < 0.05), indicating that the model had a good fit and a small experimental error, both of which are valuable for accurate predictions. Furthermore, this model was also used to predict and optimize the solid: liquid ratio, concentration of the extraction solution, and extraction time. The *p*-value showed that the primary term of factors B, AB, and BC in UAE and factor A in HAE had a significant effect on the comprehensive score (*p* < 0.05), whereas the other elements had no significant effect on the result (*p* > 0.05). As the extraction effect was better for HAE than for UAE, HAE was used for subsequent analyses. According to the established polynomial model, through Design-Export 10.0 software statistics, factors A, B, and C were drawn to create the corresponding surface map (Figure 3). The area of each factor has been optimized in the figure, and the following conditions for extracting total triterpenoids from *I. obliquus* were determined for HAE: extraction solution, [BMIM]Br with MeOH; concentration of the extraction solution, 1.0 mol/L; solid: liquid ratio, 1:30; and extraction time, 83.64 min.

### 2.3. Process Parameter Optimization and Model Validation

To verify the reliability of the model, the experiment was performed according to the optimized extraction conditions described in Section 3.2. Under the same conditions, the total triterpene amounts extracted from 1 g of *I. obliquus* were 16.81, 16.79, and 16.75 mg, respectively. Compared with the predicted value of 16.96 mg, the relative deviations were as less as 0.88, 1.00, and 1.24%, respectively, indicating that the mathematical model has good predictability.

### 2.4. Evaluation of the Potential Inhibitory Activity of LDH

**Figure 4:**
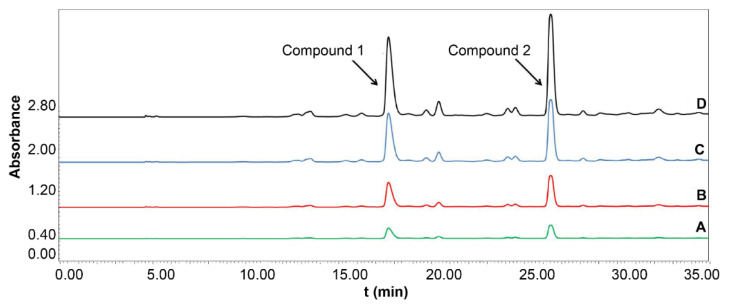
Ultrafiltration high-performance liquid chromatography chromatograms of the lactate dehydrogenase (LDH) inhibitors present in the crude sample of *Inonotus obliquus* (detection wavelength: 300 nm). (A) 20 U/mL LDH; (B) 10 U/mL LDH; (C) 5 U/mL LDH; (D) blank.

The potential LDH inhibitors in *I. obliquus* were screened using UF-LC. The enzyme-ligand complexes were separated from the small unconjugated molecules through the UF membrane. The binding of *I. obliquus* at 50 mg/mL to LDH at different concentrations was evaluated, and two compounds were considered as potential LDH ligand compounds (Figure 4). The retention time of compound 1 was 17.98 min, and the inhibition rates of 5, 10, and 20 U/mL LDH were 23.38, 39.06, and 54.58%, respectively. For compound 2, the retention time was 26.17 min, and the inhibition rates at 5, 10, and 20 U/mL LDH were 27.72, 48.45, and 69.44%, respectively. Because compounds 1 and 2 had potent inhibitory effects on LDH, HSCCC was used to separate them.

### 2.5. HSCCC Separation of Potential LDH Inhibitors from I. Obliquus

The separation effect of HSCCC mainly depends on the selection of the solvent system. In the present study, different proportions of *n*-hexane-ethyl acetate-MeOH-water were used as the solvent system. The *K* values for compounds 1 and 2, and the resolutions of both compounds, were calculated as described in Section 3.9. The *K* values of compounds 1 and 2 were calculated as *K* = A_u_/A_l_, and their resolution was the ratio of *K*_1_ to *K*_2_.

When the *K* value is in the ideal range (0.5–2.5), the greater the resolution, the better the separation. In the present study, *n*-hexane-ethyl acetate-MeOH-water (5:5:3:7) was used as the solvent system for the separation of compounds 1 and 2, with *K*_1_ = 1.29, *K*_2_ = 0.99, and a resolution of 1.30 (Table 3).

**Figure 5:**
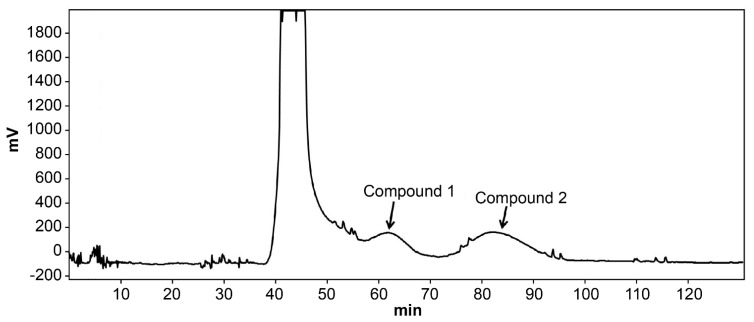
High-speed countercurrent chromatography separation of the crude extract of *Inonotus obliquus*. *n*-hexane-ethyl acetate-MeOH-water (5:5:3:7, *v*/*v*/*v*/*v*); flow rate: 1.5 mL/min; detection wavelength: 300 nm; rotational speed: 850 rpm; sample size: 100 mg.

The mobile phase flow rate determines the retention rate of the stationary phase and the separation of the chromatographic peaks. At a column rotation speed of 850 rpm and a flow rate of 1.0 mL/min, the retention rate was 72%, and the separation time of 180 min allowed compounds 1 and 2 to separate. When the flow rate was increased to 2.5 mL/min, the retention rate was 60%, and the separation time was only 100 min. Although the separation time was shortened, compound 1 could not be separated from other impurity peaks. At a flow rate of 1.5 mL/min, compounds 1 and 2 were distinctly separated within 120 min with a 68% retention rate. Hence, the HSCCC separation conditions used were as follows: column rotation, 850 rpm; solvent system, *n*-hexane-ethyl acetate-MeOH-water (5:5:3:7); flow rate, 1.5 mL/min; and injection volume, 100 mg (Figure 5).

### 2.6. Purity Determination and Identification of Potential LDH Inhibitors Using High-Performance Liquid Chromatography (HPLC), Electrospray Ionization–Mass Spectrometry (ESI–MS), ^1^H Nuclear Magnetic Resonance (NMR), and ^13^C NMR

**Figure 6:**
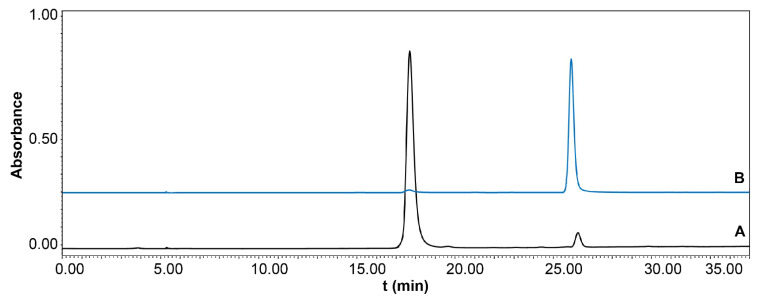
High-performance liquid chromatography chromatograms of compounds 1 and 2. (A) Compound 1, identified as betulin; (B) Compound 2, identified as lanosterol.

**Figure 7:**
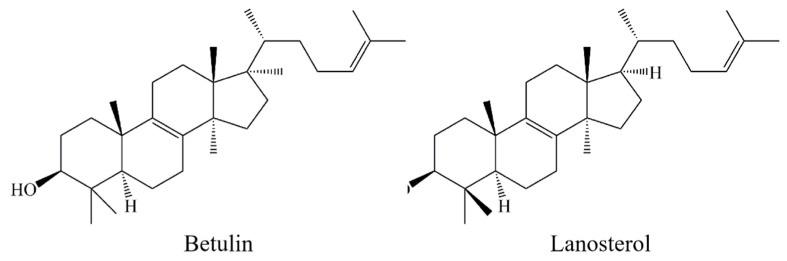
The chemical structures of betulin and lanosterol.

Potential LDH inhibitors in *I. obliquus* extracts were analyzed via HPLC. The purity of compounds 1 and 2 were 95.9% and 97.8%, respectively (Figure 6). In the positive ion mode, the mass spectra of compounds 1 and 2 were recorded. Compounds 1 and 2 were identified as betulin and lanosterol, respectively, based on their HPLC retention times, MS/MS data, as well as ^1^H and ^13^C NMR data [22,23]. The chemical structures of betulin and lanosterol are shown in Figure 7.

The study has certain limitations, which can be overcome in the future. First, since some ionic liquids are expensive, it is crucial to perform the least number of experiments to determine the best scheme of ionic liquid extraction in the extraction process. Additionally, studies have shown that long alkyl chain ILs exert toxicity; therefore, the selection and design of ILs should be carefully considered. Second, ultrafiltration liquid chromatography can only be used to detect the inhibitory enzyme activity of compounds in vitro and cannot be used to represent the medicinal effect of compounds in vivo.

## 3. Materials and Methods

### 3.1. General Experimental Procedures

A BS-124S electronic balance (Sartorius, Gottingen, Germany) was used for weight measurements. An ultraviolet-visible spectrophotometer (Thermo Fisher Scientific, San Jose, CA, USA) was used for absorbance measurements. HPLC was conducted on a Waters 2695 instrument (DAD, Milford, CT, USA) coupled to a Waters 2998 diode array detector (DAD). A Sigma 1–14 centrifuge (Sigma Zentrifugen, Osterode am Harz, Germany) equipped with an ultra-membrane filter Microcon YM–100 (Millipore, Bedford, MA, USA) was also used. ESI–MS was performed using an LCQ Fleet ion-trap mass spectrometer (Thermo Fisher Scientific, San Jose, CA, USA) equipped with an ESI source (Thermo Fisher Scientific, San Jose, CA, USA). HSCCC was performed on a TBE-300B Spectrum HSCCC (Shanghai Tauto Biotech Co., Ltd., Shanghai, China). ^1^H and ^13^C NMR spectra were recorded on a Bruker AV-400 and Avance-600 spectrometer (Bruker BioSpin, Ettlingen, Germany).

### 3.2. Chemicals and Reagents

HPLC-grade MeOH for HPLC and LC-MS was purchased from Fisher Scientific (UK). Ultra-pure water (18.2 MΩ-cm) was produced using a Milli-Q water purification system (Millipore, Burlington, MA, USA). LDH was obtained from Sigma-Aldrich (St. Louis, MO, USA), and phosphate-buffered saline (PBS) from Bueke (Switzerland). All organic solvents used for *I. obliquus* extraction and HSCCC separation were of analytical grade and purchased from Beijing Chemical Works (Beijing, China). The *I. obliquus* specimen used for extraction was stored in the Central Laboratory of Changchun Normal University, China (voucher specimen number: CCSFU-W1702). All ILs, vanillin, oleanolic acid, and acid standards were purchased from Shanghai Aladdin Biochemical Technology Co., Ltd. (Shanghai, China).

### 3.3. Determination of Total Triterpenoids

The total triterpenoid content was determined via vanillin-glacial acetic acid-perchloric acid spectrophotometry using oleanolic acid as the standard. Oleanolic acid (10 mg) was dissolved in MeOH in a 50-mL flask to prepare a 0.2 mg/mL standard solution. Standard solutions (0.0–0.7 mL) in test tubes were evaporated in a 90 °C water bath. Thereafter, 0.3 mL 5% vanillin-glacial acetic acid solution and 1 mL perchloric acid were added to each tube and mixed evenly. After 20 min in a water bath at 60 °C, mixtures were rapidly cooled to room temperature (25 °C) with cold water, diluted with glacial acetic acid in a 10-mL flask, and shaken. The absorbance (Y, ordinate) of each mixture was measure at 550 nm and plotted against the mass (X, abscissa) of oleanolic acid. The total triterpenoid content was obtained in mg oleanolic acid per gram of dried *I. obliquus*.

### 3.4. Extraction of ILs

#### 3.4.1. UAE

Seven types of ILs and four dispersants were used as extraction solutions. Four types of ILs with different carbon-chain lengths were selected: [EMIM]PF_6_, [BMIM]PF_6_, [HMIM]PF_6_, and [OMIM]PF_6_; ILs with similar cation and anion compositions were also investigated: [BMIM]TfO, [BMIM]BF_4_, [BMIM]Br, and [BMIM]PF_6_. The four dispersants were MeOH, acetonitrile (ACN), H_2_O, and acetone (ACE) (Kunshan Ultrasonic Instrument Co., Ltd., Kunshan, China). The concentration of extraction solutions (0.1–1.0 mol/L), duration of extraction (15–100 min), and solid: liquid ratio (1:10–1:30) varied for extracting triterpenoids from 1 g of *I. obliquus*. During the preliminary screening, when the solid: liquid ratio increased from 1:30 to 1:40, the total triterpenoid content of *I. obliquus* did not increase significantly. To optimize solvent usage, 1:30 was selected as the maximum solid: liquid ratio. When the concentration of the extract increased from 1.0 mol/L to 1.2 mol/L, the total triterpenoid content of *I. obliquus* decreased, and when the concentration of the extraction solution was too low, the ILs could not be fully utilized; hence, a concentration of the extraction solution range from 0.1 mol/L to 1.0 mol/L was selected. Furthermore, the total triterpenoid content of *I. obliquus* decreased slightly at 110 min and greatly after 120 and 150 min [24]. UAE was performed using a 750 W ultrasonic processor (BS-250; Kunshan Ultrasonic Instrument, Co., Ltd., Kunshan, China) at 80% amplitude [25] and 31 °C. Because ultrasonic waves increase the solvent temperature, ice coating was used to control the temperature during the extraction process. The extracts were centrifuged at 10,000× *g* for 5 min; then, 0.1 mL of the supernatant was evaporated until dryness in a water bath at 60 °C, and a post-treatment test was conducted as described below. All the samples were prepared and analyzed in triplicate [26].

#### 3.4.2. HAE

Total triterpenoids were also extracted from *I. obliquus* via HAE, and the factor level was consistent with that of UAE. In our preliminary screening, the total triterpenoid yield gradually increased with increasing temperature; therefore, 70 °C was selected as the HAE temperature [27] and the extract treatment was the same as that used for UAE.

### 3.5. Process Level Screening

#### 3.5.1. Single-Factor Experiment

##### Effect of IL Type on Total Triterpenoid Extraction

When the solid: liquid ratio was 1:10, the concentration of the extraction solution was 0.6 mol/L, extraction time was 80 min, and dispersant was MeOH. Different extraction solutions containing seven types of ILs ([BMIM] TfO, [BMIM] BF4, [BMIM] Br, [BMIM]PF_6_, [EMIM]PF_6_, [HMIM]PF_6_, and [OMIM]PF_6_) were examined. The index of total triterpenoids per gram of *I. obliquus* was evaluated in all experiments. The extraction ability of different IL components in UAE and HAE was evaluated. The highest extraction yield was obtained by using [BMIM] Br. All samples were prepared and analyzed in triplicate.

##### Effect of Dispersant Type on Total Triterpenoids

Using 1 g of *I. obliquus* at a 1:10 solid: liquid ratio, the concentration of the extraction solution was 0.6 mol/L, extraction time was 80 min, and IL was [BMIM]Br. The extraction ability of different dispersants (ACN, MeOH, H_2_O, and ACE) was investigated using UAE and HAE. The results showed that when the dispersant was MeOH, the extraction yield was the highest.

#### 3.5.2. Optimization of the Central Composite Design

*I. obliquus* (1 g) was extracted with a solution composed of [BMIM] Br as the IL and MeOH as the dispersant. Different solid: liquid ratios (1:10–1:30), concentration of the extraction solution (0.1–1.0 mol/L), and extraction times (15–100 min) were evaluated using the central composite design. There were five levels for each factor. The minimum level was set as −1.682 and the maximum as +1.682; other levels (−1, 0, and +1) were calculated using Equation (1).

(1) $$\frac{-1-\left(-11.682\right)}{x-10}=\frac{1.682-\left(-1.682\right)}{30-10}.$$

For example, for factor A: −1.682 is 10 and +1.682 is 30; if the level of A is −1, the value is x, and the value of x is obtained by Equation (1). The levels of the different factors are shown in Table 4.

The total triterpenoid content in each experimental group was determined using the central composite design, and the experimental data for each group were fitted and analyzed. The experimental scheme and results are shown in Table 1. The same experimental conditions were repeated six times to verify the central point of the experiment, and the results were used to investigate the error of the model. Using Design-Expert 10.0 software (Stat-Ease, Minneapolis, MN, USA) for UAE and HAE statistical analyses, the effect surface map of the two factors in the model was drawn to each evaluation index. The model often reflected the change in the response value with a smaller error, had a significant impact, and better analyzed and predicted the extraction process, based on which optimal scheme was selected.

### 3.6. Statistical Analysis

Design-Expert 10.0 software was used to analyze the extraction conditions of triterpenoids from *I. obliquus*.

### 3.7. Determination of the Potential Inhibitory Activity of LDH

The potential LDH-inhibitory activity of *I. obliquus* was determined by UF-LC. The ultrafiltration process included culture, washing, and separation [28]. *I. obliquus* extract (50 mg) was dissolved in 50% MeOH (1 mL) and filtered using a 0.45-μm membrane. The filtrate (10 μL; 50 mg/mL), PBS (pH 7.4; 100 μL), and LDH (90 μL; 5, 10, and 20 U/mL) were mixed evenly. LDH was replaced with 90 μL PBS in the blank solution. All the four groups (blank, 5, 10, and 20 U/mL LDH) were incubated at 37 °C for 30 min. The mixture was then centrifuged at 10,000× *g* and 25 °C for 10 min with an ultrafiltration membrane (molecular weight cut-off, 100 kDa) to remove any unbound compounds. Thereafter, 100 μL MeOH: water mixture (50:50, *v*/*v*, pH 3.3) was added to the UF membrane and centrifuged at 10,000× *g* for 10 min (×3) to release the bound ligand [29]. The LDH inhibition rate was calculated using the equation (Aa−Ab)/Aa × 100%, where Aa and Ab are the absorbances of the blank and the experimental group, respectively [30]. Finally, the solvent was removed under vacuum, and the released ligand was used for further analyses.

### 3.8. High-Performance Liquid Chromatography-Diode Array Detector–Mass Spectrometry (HPLC–DAD–MS) Analysis

The HPLC–DAD–MS technique was used to analyze the *I. obliquus* extract. A Waters 2695 C_18_ column (250 × 4.6 mm, 5 μm) was used with a MeOH (A) and water (B) mobile phase at a 0.5 mL/min flow rate. The elution procedure was as follows: 0–30 min, 24–56% solvent A; 30–35 min, 56–76% solvent A. Online ultraviolet spectra were detected at 300 nm. An LCQ Fleet ion-trap mass spectrometer was connected to the photo-diode array instrument by an ESI interface and used to perform the MS and MS^n^ analyses. The mass spectrometer was operated in the positive ion mode, and the flow rate was 0.15 mL/min. The capillary voltage was set at −20 V in the positive ion mode. A full scan was performed using selected reaction monitoring mode-based identification at 4.5 kV spray voltage, 350 °C capillary temperature, and 10 psi pressure. A 100–1200 m/z scan range at a resolution of 17,500 was employed.

### 3.9. Separation of LDH Inhibitors by HSCCC

According to the partition coefficient (*K*) and the resolution of target compounds (compounds 1 and 2), the HSCCC two-phase solvent system was selected. The two-phase solvent system was thoroughly mixed at 25 °C; then, 10 mg of *I. obliquus* extract was added, and 2 mL of each of the upper and lower phases were collected. After the solvent was evaporated, the phases were analyzed using HPLC. Under the same operating conditions, the peak areas of the upper and lower phases were recorded as A_u_ and A_l_, respectively. The *K* values of compounds 1 and 2 were calculated as *K* = A_u_/A_l_, and their resolution was the ratio of *K*_1_ to *K*_2_.

For this study, the stationary phase was the upper phase, and the mobile phase was the lower phase. During each separation process, the coiled column was filled with the stationary phase. Following column rotation at 850 rpm for 30 min, the mobile phase was pumped into the column at a flow rate of 1.5 mL/min. When the hydrodynamic equilibrium was reached, the sample solution (100 mg) was injected. The detection wavelength was set at 300 nm, and the eluted compound was collected in the fraction collector. The solvents were then removed from the collected components using a rotary evaporator and analyzed. Following the elution of the required peaks (compounds 1 and 2), the program was stopped, and the stationary phases remaining on the column were collected. It should be noted that the retention rate of the stationary phase is equal to the volume of the stationary phase divided by the total volume of the chromatographic column.

### 3.10. Analytical Data for Compounds 1 and 2

The main features of compounds 1 and 2 are summarized in Table 5.

## 4. Conclusions

The total triterpenoids in *I. obliquus* were extracted in UAE and HAE modes using ILs, different anions, and dispersants. [BMIM]Br with MeOH served as the extraction solution for subsequent analyses. The solid:liquid ratio, concentration of the extraction solution, and extraction time were optimized using the central composite design and verified. The concentration of the extraction solution was 1.0 mol/L, the solid:liquid ratio was 1:30, the extraction time was 83.64 min, and the temperature was 70 °C, in which the highest total triterpene content was obtained from *I. obliquus* using HAE. Potential LDH inhibitors in *I. obliquus* were screened via UF-LC-MS and identified as betulin and lanosterol, which were then separated via HSCCC within 120 min with purities of 95.9% and 97.8%, respectively. Thus, we demonstrated that IL-HAE could be applied for triterpenoid extraction from *I. obliquus*, and UF-LC-MS is a fast and feasible method for studying the potential medicinal value of these compounds.

## Figures and Tables

**Table 1:** Central composite design and indicator results.

No.	Factor			Total Triterpenoids (mg)	
	A	B	C	UAE	HAE
1	0	0	0	8.708	10.090
2	+1.682	0	0	8.500	15.446
3	0	0	0	8.708	10.090
4	0	−1.682	0	4.285	12.301
5	−1	−1	−1	8.271	9.147
6	0	0	0	8.708	10.090
7	0	+1.682	0	8.846	12.716
8	−1.682	0	0	6.220	9.537
9	+1	+1	−1	7.523	10.569
10	−1	−1	+1	7.492	7.979
11	−1	+1	+1	9.536	9.147
12	−1	+1	−1	7.590	7.395
13	0	0	+1.682	6.911	10.781
14	+1	+1	+1	11.823	11.823
15	0	0	0	8.708	10.090
16	+1	−1	+1	5.016	9.852
17	0	0	0	8.708	10.090
18	0	0	0	8.708	10.090
19	+1	−1	−1	6.270	10.569
20	0	0	−1.682	5.943	7.602

A: solid-liquid ratio; B: concentration of the extraction solution; C: extraction time.

**Table 2:** Analysis of variance of the quadratic multiple regression equation.

Source	Sum of Squares	Df	Mean Square	F Value	*p*	Significant
**UAE**						
Model	45.82	9	5.09	5.35	0.0075	Significant
A–Solid:liquid ratio	0.18	1	0.18	0.19	0.6711	
B–Concentration of the extraction solution	21.4	1	21.4	22.47	0.0008	
C–Extracting time	2.5	1	2.5	2.62	0.1363	
AB	5.61	1	5.61	5.89	0.0357	
AC	0.44	1	0.44	0.46	0.5114	
BC	8.57	1	8.57	9	0.0133	
A^2^	0.62	1	0.62	0.66	0.4372	
B^2^	3.45	1	3.45	3.62	0.0863	
C^2^	4.17	1	4.17	4.38	0.0628	
Residual	9.52	10	0.95			
Lack of Fit	9.52	5	1.9			
Pure Error	0	5	0			
Cor Total	55.34	19				
Fitting equations	Y = 8.67 + 0.12A + 1.25B + 0.43C + 0.84AB + 0.23AC + 1.03BC − 0.21A^2^ − 0.49B^2^ − 0.54C^2^ (R^2^ = 0.8279)					
**HAE**						
Model	48.57	9	5.4	3.54	0.0308	Significant
A—Solid-liquid ratio	26.66	1	26.66	17.49	0.0019	
B—Concentration of the extraction solution	0.32	1	0.32	0.21	0.6575	
C—Extracting time	3.06	1	3.06	2.01	0.1868	
AB	0.82	1	0.82	0.54	0.4812	
AC	2.76 × 10^−4^	1	2.76 × 10^−4^	1.81 × 10^−4^	0.9895	
BC	2.99	1	2.99	1.96	0.1916	
A^2^	2.85	1	2.85	1.87	0.2015	
B^2^	2.93	1	2.93	1.92	0.196	
C^2^	7.51	1	7.51	4.93	0.0507	
Residual	15.24	10	1.52			
Lack of Fit	15.24	5	3.05			
Pure Error	0	5	0			
Cor Total	63.81	19				
Fitting equations	Y = 10.15 + 1.40A + 0.15B + 0.47C + 0.32AB − 5.875 e^− 003^AC + 0.61BC + 0.44A^2^ − 0.45B^2^ − 0.72C^2^ (R^2^ = 0.7611)					

HAE, heat-assisted extraction; UAE, ultrasonic-assisted extraction.

**Table 3:** K values of *Inonotus obliquus* in different two-phase solvent systems composed of *n*-hexane-ethyl acetate-MeOH-water.

Solvent System (*v*/*v*)	Compound 1 (K_1_)	Compound 2 (K_2_)	K_1_/K_2_
1:1:1:1	0.42	0.85	0.49
5:5:3:7	1.29	0.99	1.30
1:1:2:2	7.58	2.73	2.78

**Table 4:** Factors and levels of the central composite design.

Factor	Level				
	−1.682	−1	0	+1	+1.682
A (solid-liquid ratio)	10	14.055	20	25.945	30
B (concentration of the extraction solution, mol/L)	0.1	0.282	0.55	0.818	1
C (extracting time, min)	15	32.232	57.5	82.767	100

**Table 5:** Main features of compounds 1 and 2.

	Compound 1	Compound 2
Name	Betulin^22^	Lanosterol^23^
Retention time-t_R (min)_	17.98	26.17
ESI–MS (+) m/z	442 [M]^+^	426 [M]^+^
Molecular formula	C_30_H_50_O	C_30_H_50_O
^1^H-NMR (400 MHz, CDCl_3_) δ	0.75 (3H, s, H-24), 0.82 (3H, s, H-25), 0.96 (3H, s, H-23), 0.97 (3H, s, H-27), 1.02 (3H, s, H-26), 1.68 (3H, s, H-30), 3.18 (1H, dd, J = 11.2, 4.8 Hz, H-3), 3.31 (1H, d, J = 10.8 Hz, H-28α), 3.78 (1H, d, J = 10.8 Hz, H-28β), 4.58 (1H, s, H-29α), 4.68 (1H, s, H-29β)	0.68 (3H, s, H-30), 0.80 (3H, s, H-18), 0.87 (3H, d, J = 6.5 Hz, H-21), 0.90 (3H, s, H-28), 0.99 (3H, s, H-19), 1.03 (3H, s, H-29), 1.60 (3H, s, H-27), 1.68 (3H, s, H-26), 3.21 (1H, dd, J = 3.4, 9.3 Hz, H-3), 5.08 (1H, t, J = 5.2 Hz, H-24)
^13^C-NMR (100 MHz, CDCl_3_) δ	38.6 (C-1), 27.3 (C-2), 79.0 (C-3), 38.8 (C-4), 55.2 (C-5), 18.3 (C-6), 34.2 (C-7), 40.9 (C-8), 50.3 (C-9), 37.1 (C-l0), 20.8 (C-11), 25.1 (C-12), 37.2 (C-13), 42.7 (C-14), 27.0 (C-15), 29.1 (C-16), 47.7 (C-17), 47.7 (C-18), 48.7 (C-19), 150.5 (C-20), 29.7 (C-21), 33.9 (C-22), 27.9 (C-23), 15.3 (C-24), 16.1 (C-25), 15.9 (C-26), 14.7 (C-27), 60.5 (C-28), 109.7 (C-29), 19.0 (C-30)	36.3 (C-1), 28.2 (C-2), 79.0 (C-3), 38.9 (C-4), 50.9 (C-5), 21.0 (C-6), 27.8 (C-7), 134.3 (C-8), 134.3 (C-9), 37.0 (C-10), 18.2 (C-11), 26.5 (C-12), 44.4 (C-13), 49.8 (C-14), 30.9 (C-15), 30.8 (C-16), 50.3 (C-17), 15.4 (C-18), 18.6 (C-19), 36.2 (C-20), 19.1 (C-21), 35.3 (C-22), 25.7 (C-23), 125.2 (C-24), 130.9 (C-25), 24.9 (C-26), 17.6 (C-27), 24.2 (C-28), 27.9 (C-29), 15.7 (C-30)

## Data Availability

The data presented in this study are available in the published article.

## Sample Availability

Samples of the compounds are not available from the authors.
